# Characterization of brain-wide somatosensory BOLD fMRI in mice under dexmedetomidine/isoflurane and ketamine/xylazine

**DOI:** 10.1038/s41598-021-92582-5

**Published:** 2021-06-23

**Authors:** Taeyi You, Geun Ho Im, Seong-Gi Kim

**Affiliations:** 1 Center for Neuroscience Imaging Research (CNIR), Institute for Basic Science (IBS), Suwon, 16419 South Korea; 2 Department of Biomedical Engineering , Sungkyunkwan University, Suwon, 16419 South Korea; 3 Department of Intelligent Precision Healthcare Convergence, Sungkyunkwan University, Suwon, 16419 South Korea

**Keywords:** Neuroscience, Physiology

## Abstract

Mouse fMRI under anesthesia has become increasingly popular due to improvement in obtaining brain-wide BOLD response. Medetomidine with isoflurane has become well-accepted for resting-state fMRI, but whether this combination allows for stable, expected, and robust brain-wide evoked response in mice has yet to be validated. We thus utilized intravenous infusion of dexmedetomidine with inhaled isoflurane and intravenous infusion of ketamine/xylazine to elucidate whether stable mouse physiology and BOLD response are obtainable in response to simultaneous forepaw and whisker-pad stimulation throughout 8 h. We found both anesthetics result in hypercapnia with depressed heart rate and respiration due to self-breathing, but these values were stable throughout 8 h. Regardless of the mouse condition, brain-wide, robust, and stable BOLD response throughout the somatosensory axis was observed with differences in sensitivity and dynamics. Dexmedetomidine/isoflurane resulted in fast, boxcar-like, BOLD response with consistent hemodynamic shapes throughout the brain. Ketamine/xylazine response showed higher sensitivity, prolonged BOLD response, and evidence for cortical disinhibition as significant bilateral cortical response was observed. In addition, differing hemodynamic shapes were observed between cortical and subcortical areas. Overall, we found both anesthetics are applicable for evoked mouse fMRI studies.

## Introduction

Functional magnetic resonance imaging (fMRI) has become a valuable tool for the indirect investigation of brain activity. fMRI relies on the coupling of neural activity to vascular hyperemia, termed neurovascular coupling, which recruits excessive oxygenated blood in the active areas leading to increased blood oxygenation-level dependent (BOLD) contrast^1^. In humans, fMRI has been well utilized in studying cognitive functions in response to a task or detecting functionally connected network differences between healthy and diseased brains during a task-free resting state scan. The use of fMRI has branched to rodents for the prospect of combining transgenic, pharmacological, or surgical modifications with in-vivo imaging to probe more sophisticated neuroscience questions^2^. Improvements in magnetic field strength has helped increase the fMRI signal obtainable from the small rodent brain since the BOLD effect increases linearly with field strength^3^. Similarly, improvements in coils, which detect the signal, such as the cryogenically cooled volume coil helps to increase the signal to noise ratio^4,5^. These improvements have led to a growth of successful rodent fMRI experiments that has supplemented translational brain research^6–11^.

However, a key difference between rodent and human fMRI is the use of anesthesia to immobilize the rodents while scanning, which clouds interpretation within rodent fMRI results and between rodents and humans. As the animals are not voluntary subjects, anesthesia is used to mount the rodent head ethically and painlessly since motion is detrimental to image quality and can induce false signals. However, anesthesia leads to alterations in brain state, cardiovascular physiology, and hemodynamic properties which all affect the BOLD signal^12–14^. Awake rodent fMRI has been successfully conducted, yet this limits the duration and type of study capable of being performed and requires stringent habituation and monitoring for stress^7,15,16^. Therefore, the use of anesthesia is still practical for rodent fMRI. Consequently, researchers have worked to elucidate various anesthetics’ effect on hemodynamics and neural activity in rats and mice to interpret BOLD fMRI results. Contralateral responses to single-paw stimulation are consistently observed in rats under various anesthetics including α-chloralose^17^, isoflurane^18^, and medetomidine^19^, yet many mouse fMRI to single-paw stimulation showed either widespread bilateral fMRI responses, indicating arousal-related hemodynamic responses, or low sensitivity^20–23^. Although both belong to the Rodentia order, there are pharmacological, behavioral, and developmental differences between mice and rats which may explain differences in anesthetic effect^24^. Thus, despite the plethora of successful rat fMRI studies, caution must be taken in interpreting the results with mice for translational neuroscience. In addition, recombinant transgenic mouse models still outnumber those of rats, thus systematic investigation of mouse fMRI is critically important for evaluating the feasibility to combine the various transgenic models with fMRI. To date, two anesthetic combinations, dexmedetomidine/isoflurane and ketamine/xylazine have been repeatedly and successfully used for evoked mouse fMRI studies.

Medetomidine is an α2 agonist that induces sleep-like sedation by inhibiting arousal nuclei in the locus coeruleus, and has become a promising choice owing to its well documented properties, particularly in rats^23,25–27^. It is a racemic mixture with dexmedetomidine being the active component^26^. Although it leads to vasoconstriction, which can impair BOLD response, it has been shown to preserve vasoreactivity and can be quickly reversed by administration of atipamezole leading to its favorable application with fMRI^27,28^. Multiple rat fMRI studies under medetomidine report longitudinal robust cortical and thalamic response from electrical forepaw stimulation along with its applicability in resting state connectivity^19,29–33^. Several years after the first rat application^19^, Adamczak *et al.*^34^ introduced its implementation in mice, yet only reported cortical response to electrical forepaw stimulation. In addition, sedation duration was limited to 120 minutes while 210 to 360 min were reported in rats^30^. Nasrallah *et al*.^35^ expanded the use of medetomidine to show observable resting state networks and reported that the BOLD response from forepaw stimulation is not affected by dose. Both these studies failed to show thalamic activity which may be attributed to a higher medetomidine concentration and/or self-breathing condition. Thalamic activity was detected in ventilated studies, however, bilateral cortical activity to single-paw stimulations were reported which was attributed as influence from the arousal response possibly due to the combination of electrical stimulation and intubation^23,36^. Currently, medetomidine in combination with inhaled GABA_A_ agonist isoflurane is used in resting state fMRI studies due to its property in preserving translatable brain-wide functional connectivity^33,37–39^, along with visual^40^ and olfactory^41^ evoked fMRI in mice. The use of a balanced multimodal anesthesia allows for the use of a lower dose of each individual anesthetics thus reducing its effect on its respective mode of action^5^. In addition, a multimodal approach allows for counterbalancing the physiological and hemodynamic effect of each anesthetic which may allow for a more normal physiological and hemodynamic state^25,37,42^. However, there is lacking validation if the combination allows for expected brain-wide evoked response as the aforementioned studies only presented with thalamic and primary cortical response without higher-order or association areas. In addition, it is currently unknown if the combination preserves expected somatosensory activation in mice.

Alternative to medetomidine anesthesia, we previously developed an intermittent intraperitoneal ketamine/xylazine (K/X) anesthesia protocol for mouse fMRI^43^ with several subsequent mouse fMRI studies utilizing ketamine or the cocktail^9,16,41,44^. The cocktail is commonly used during animal surgery, but when applied with fMRI, we found it to have robust, longitudinal cortical response to forepaw stimulation at 9.4 Tesla. Weak thalamic activity was also detected suggesting higher sensitivity compared to medetomidine, with robust activity appearing at 15.2 Tesla^45^. Similar to medetomidine/isoflurane, a multimodal approach is used to counterbalance each anesthetic’s effect. Xylazine is also an α2 agonist, but weaker than medetomidine^46,47^. Ketamine is an NMDA receptor antagonist that primarily act on cortical inhibitory and excitatory neurons resulting in increased recurrent excitation^48,49^. This, along with our previous fMRI studies, suggests K/X to be a valid anesthetic for mouse fMRI compared to medetomidine due to the higher sensitivity observed in mouse. However, ketamine is also known to have psychoactive effects that may introduce spurious activity on the brain which may not be ideal for functional studies. Thus, to validate K/X further, we aim to test if brain-wide and expected functional response is preserved under K/X in comparison to dexmedetomidine/isoflurane (D/I).

We propose to examine the detectability and stability of mouse fMRI response to somatosensory stimulation under D/I anesthesia, with a protocol similarly used for resting state, and K/I at an ultrahigh field of 15.2 Tesla. To initially address whether intravenous (IV) infusion of D/I or K/X is viable for evoked fMRI studies in mice, we first observed arterial blood gases over an eight-hour infusion period on the bench. Then, under continuous IV infusion of both anesthetics, BOLD fMRI responses were evaluated for sensitivity and stability during simultaneous electrical forepaw (FP) and whisker-pad (WP) stimulation throughout eight hours. We found both anesthetics resulted in stable and expected brain-wide response to WP and FP stimulation albeit with differences in sensitivity, spread, and dynamics of the BOLD response. By comparing responses, we show distinct anesthetic effects that may provide better understanding of their effect on neurovascular coupling.

## Results

### Mouse physiology under IV infusion

**Figure 1:**
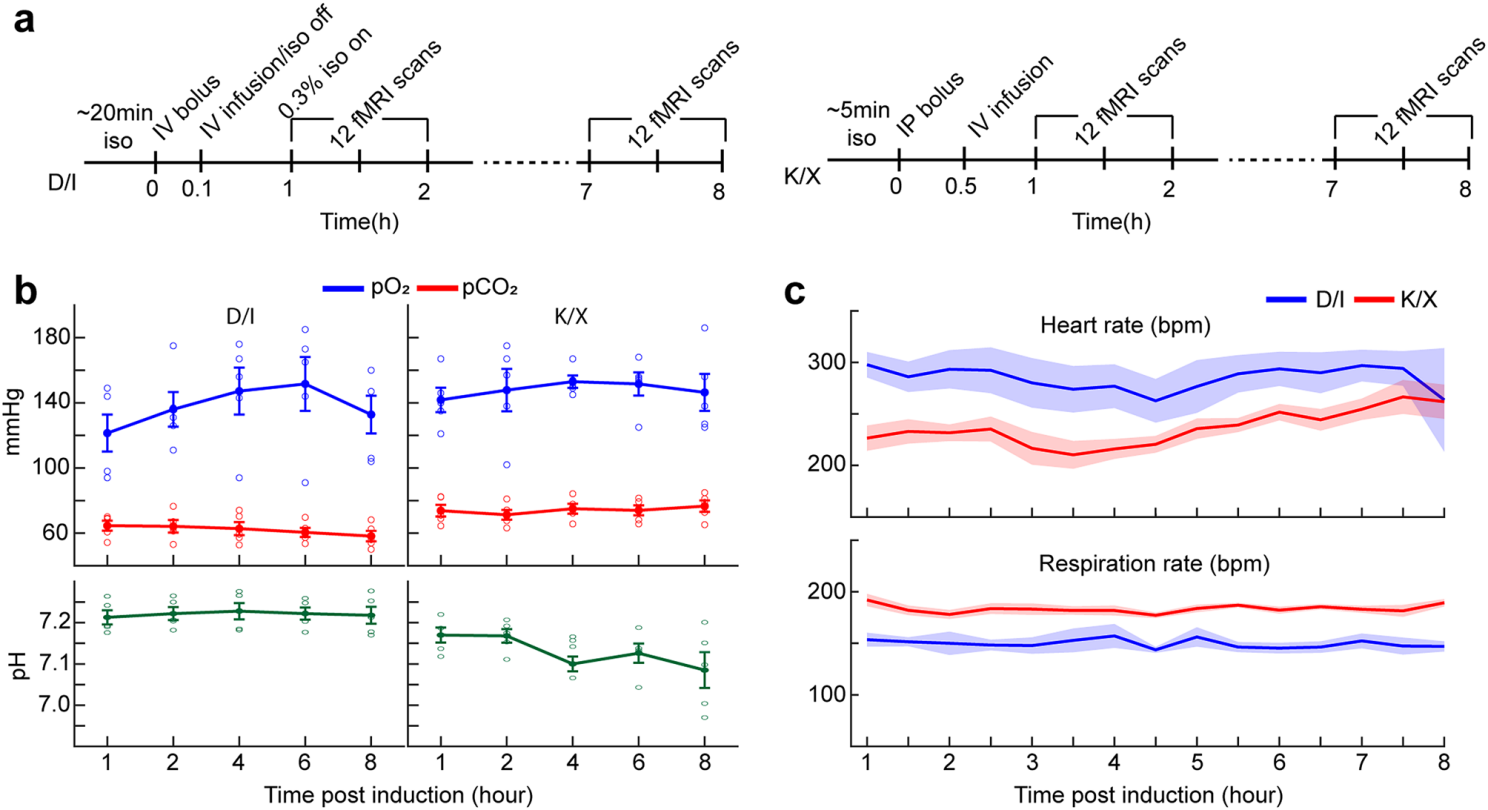
Anesthesia protocol results in sustainable physiology. (**a**) Timeline of anesthesia induction and experiment using dexmedetomidine/isoflurane (left) and ketamine/xylazine (right). (**b**) Arterial blood gas (ABG) measurements carried out 1, 2, 4, 6, and 8 h post induction shown as mean ± SEM (*n* = 5 mice per anesthetic). Each open circle represents the individual animal value. One-way ANOVA measures for (K/X;D/I): pO_2_ (*P* = 0.92;0.52), pCO_2_ (*P* = 0.83;0.65), pH (*P* = 0.17;0.98). (**c**) Heart rate and respiration rate recorded at 30 min intervals during ABG measurement. Data are represented as mean ± SEM.

Anesthesia protocol under spontaneous breathing was optimized, based on extensive preliminary studies (Fig. 1a). An IV bolus of 0.05 mg/kg of dexmedetomidine to the tail vein was followed by IV infusion of 0.05 mg/kg/h, whereas an IP bolus of ketamine/xylazine of 100 mg/kg/10 mg/kg was followed by IV infusion at a dose of 45/2.25 mg/kg/h. To verify that IV infusion of both anesthesia lead to stable animal conditions, arterial blood gas (ABG) measurements of pO_2_, pCO_2_, and pH were conducted throughout eight hours. Under D/I, mean values at start and end time point measurements for pO_2_ are 121.4 ± 11.36 (SEM) and 132.8 ± 11.58 mmHg, for pCO2 are 64.6 ± 3.09 and 58.2 ± 3.22 mmHg, and for pH are 7.21 ± 0.02 and 7.22 ± 0.02. Under K/X, mean values for pO_2_ are 141.8 ± 7.53 and 146.4 ± 11.32 mmHg, for pCO_2_ are 73.8 ± 3.62 and 76.6 ± 3.46 mmHg, and for pH are 7.17 ± 0.02 and 7.09 ± 0.04 (Fig. 1b). Two-way ANOVA showed no significant differences within anesthetics, but significant difference for pCO_2_ (*P* = 1.15e−6) and pH (*P* = 5.16e−7) between D/I and K/X were found. Interaction effects between anesthetics and time points were not significant (*P* = 0.28 for both). Mean pCO_2_ and pH averaged throughout the eight hours are 62.06 ± 0.54 mmHg and 7.22 ± 0.001 under D/I, and 74.14 ± 0.39 mmHg and 7.13 ± 0.01 under K/X. Compared to awake ABG measurements in C57BL/6 mice, both anesthesia conditions show elevated pCO_2_ and decreased pH^50^. Heart rate (HR) and respiration rate (RR) were also recorded at 30 min intervals during ABG measurements and are plotted in Fig 1c. Under D/I, no significant differences were determined by ANOVA for both HR and RR (*P* = 0.99 for both). Under K/X, significant differences were found between time points for HR (ANOVA: *P* = 0.02), while post-hoc Tukey’s multiple comparison failed to show significance with greatest difference found between 3.5 h and 7.5 h (*P* = 0.06). RR resulted in no significant difference between time points (ANOVA: *P* = 0.65). Mean HR and RR averaged throughout the eight hours are 284 ± 2.98 and 150 ± 1.04 bpm under D/I, and 236 ± 4.40 and 183 ± 1.00 bpm under K/X. Both values for both anesthetics are within range with previous findings in mice^35,51^. Although depressed when compared to awake state, these findings suggest IV infusion leads to stable physiology for both anesthetics.

### Stability and sensitivity of somatosensory-induced fMRI under D/I and K/X

**Figure 2:**
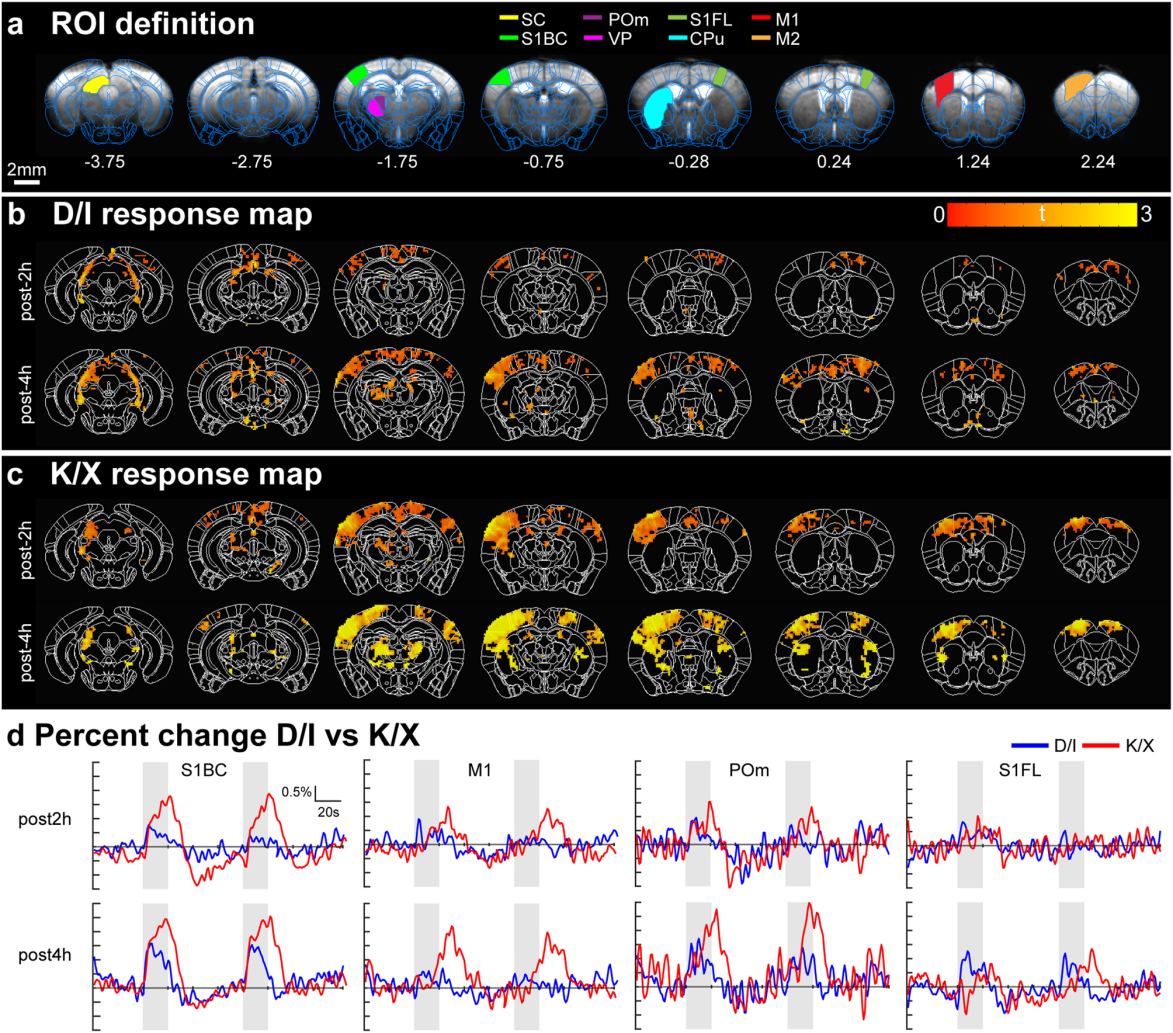
Individual mouse BOLD fMRI temporal sensitivity obtained under D/I and K/X anesthesia. (**a**) ROI definitions overlaid onto mean EPI images. CPu, caudate putamen; M1, primary motor cortex; M2, secondary motor cortex; POm, posterior medial thalamus; S1BC, primary somatosensory barrel cortex; S1FL, primary somatosensory forelimb; SC, superior colliculus; VP, ventral posterior nucleus. Coordinates below refer to distance to bregma (mm). (B-C) Response map from simultaneous FP and WP stimulation from an individual mouse under D/I (**b**) and K/X (**c**) anesthesia at 2 h and 4 h post-induction. *P* < 0.005 uncorrected. (**d**) Percent change plotted at post-2 h and post-4 h induction. Gray shaded areas indicate stimulation period. Left hemisphere ROIs were used for S1BC, S2, and POm, whereas S1FL is from the right hemisphere.

Since the IV infusion protocol resulted in stable physiology, we examined the reliability of functional responses for eight hours under both anesthetics for evoked fMRI studies with 0.13 x 0.13 mm^2^ in-plane resolution, 18 0.5-mm-thick slices, and 1 s temporal resolution. Left FP and right WP were simultaneously stimulated for maximal fMRI response, with 12 scans acquired per hourly block. Each scan consisted of 40s baseline-20s stimulation-60s interstimulus interval-20s stimulation-60s recovery. In a pilot study, we initially found FP stimulation alone resulted brain activity limited to the primary somatosensory cortex and thalamus for both anesthetics, consistent with previous findings^19,45^, and thus added WP stimulation for maximal brain activity, as the whisker somatosensory is one of the most well-developed and documented sensory regions in rodents. The mean EPI images with atlas overlay and ROI definitions are shown in Fig. 2a. To show functional sensitivity within typical experimental durations, BOLD response from a representative mouse post 2- and 4-hours induction under D/I or K/X anesthesia are shown in Fig. 2b,c. At post 2-hours induction under D/I, we detected low sensitivity in whisker-related regions contralateral to stimulated site, such as primary somatosensory barrel cortex (S1BC), primary motor cortex (M1), posterior medial thalamus (POm) and primary somatosensory forelimb (S1FL), with sensitivity increasing at post 4 hours (Fig. 2b,d). Conversely, K/X resulted in high sensitivity at post 2 hours with sensitivity increasing further at post 4 hours (Fig. 2c,d). S1FL response was weak for both anesthetics at both time points for these two mice (Fig. 2c).

Dynamic responses to stimulation were compared under both anesthetics (Fig. 2d). When comparing S1BC signal change, rapid signal increase right after the stimulus onset was similarly observed for both anesthetics. The evoked response under D/I peaked around 5 s after stimulus onset, then slightly decreased over the remaining 20 s stimulation period, while the peak was observed close to the end of stimulation after a prolonged rise under K/X. At the end of stimulation, fMRI signals recovered quickly under D/I, whereas it slowly recovered under K/X, (Fig. 2d). This property of faster hemodynamic response function under D/I, but higher amplitude under K/X is seen in all other main sensory ROIs (see Supplementary Fig. S2 online).

**Figure 3:**
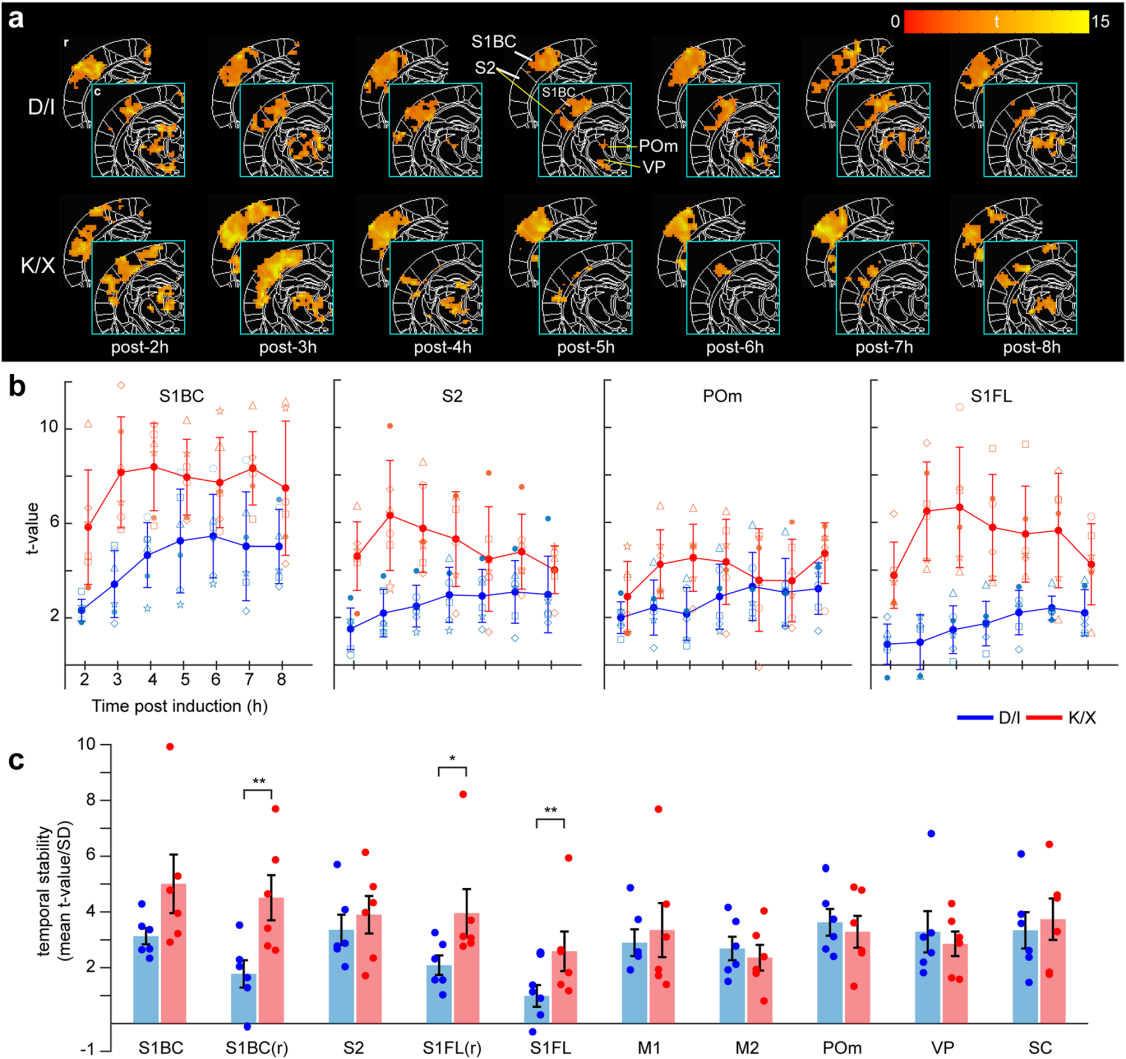
Temporal sensitivity and stability of mouse BOLD fMRI under D/I and K/X anesthesia. (**a**) Cropped group activation map showing S1BC and S2 ROIs (rostral slice) and S1BC, S2, and thalamic ROIs (caudal slice in cyan) over time under both anesthesia conditions from simultaneous FP and WP stimulation. Cluster-wise FWE corrected *P* = 0.05. r, rostral; c, caudal. (**b**) Mean t-values extracted from respective ROIs plotted over time as mean ± SD under D/I (blue) and K/X (red). Individual mouse values are plotted as different symbols (n = 6). (**c**) Temporal stability calculated by the average of t-values across time divided by SD for each mouse is plotted as mean ± SEM from each ROI. Dots represent individual mouse values (n = 6 for each anesthetic). Corrected two-sample unpaired t-test, *P < 0.05, **P < 0.01, ***P < 0.001.

To observe time-dependent BOLD stability, two slices of group-averaged fMRI maps were selected and their contralateral hemisphere to WP stimulation was displayed over 8 hours (Fig. 3a). S1BC response is consistent across both slices under D/I, whereas under K/X, S1BC response disappears in the caudal slice (outlined in cyan) while it is robust in the rostral slice. Thalamic activity seems to disappear under K/X at post-5h until post-7h which may be attributable to a high threshold and larger variance. To quantify the temporal changes, we plotted t-values from S1BC (rostral), S2 (secondary somatosensory area), POm, and S1FL (not shown in figure) over time to examine dynamic change in sensitivity for both anesthetic groups (Fig. 3b). D/I anesthesia appears to have increasing t-values over time, albeit small, while K/X values appear to decrease post 4-hours. Under K/X, we detected higher sensitivity, albeit with greater variability amongst mice, in all ROIs compared to D/I. One-way ANOVA analysis showed insignificant differences between time points for all areas and anesthesia except for S1BC under D/I (post 2h vs. 6h, *P* = 0.02, Tukey’s multiple comparison). Next, we measured the stability of the t-value by averaging across time for each mouse and dividing the mean by the standard deviation to calculate the regional temporal t-value (Fig. 3c). In general, we see sensory cortical ROIs to be more sensitive and stable under K/X than D/I except for S1BC (*P* = 0.056) and S2 (*P* = 0.54). Motor and thalamic ROIs appear to be similar between D/I and K/X. From this, we can conclude the more sensitive cortical response under K/X is stable over time, with other sensory ROIs being similar between D/I and K/X.

### Somatosensory mapping under D/I and K/X

**Figure 4:**
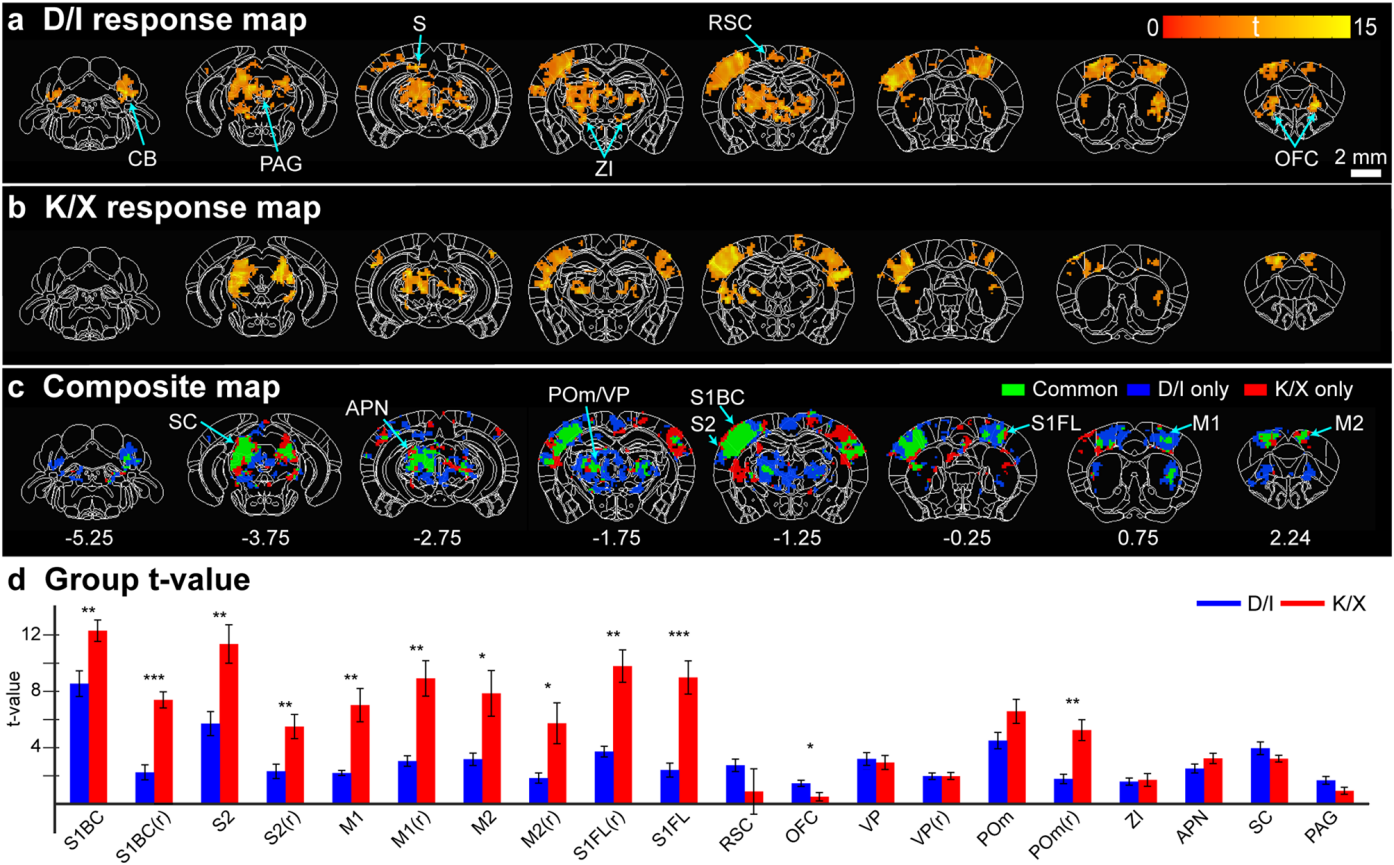
Group response map and sensitivity under D/I and K/X anesthesia (**a**–**b**) Group activation map after averaging all eight hours of runs under D/I (**a**) and K/X (**b**), cluster-wise FWE corrected *P* = 0.05. Labels in (**a**) point to clusters that appear under D/I, but not under K/X. OFC, orbital frontal cortex; RSC, retrosplenial cortex; ZI, zona incerta; S, subiculum; PAG, periaqueductal gray; CB, cerebellum. (**c**) Binarized response maps from (**a**) and (**b**) were summed to find commonly and exclusively significant clusters under both anesthesia. Coordinates below refer to distance from bregma in mm. (**d**) Group t-values extracted from anatomical ROIs based on the common and exclusive clusters from (**c**). Letter *r* in parenthesis means the right hemisphere responding to contralateral forepaw and ipsilateral whisker-pad stimulation. Data are mean ± SEM. Corrected two-sample t-test, *P < 0.05, **P < 0.01, ***P < 0.001.

**Figure 5:**
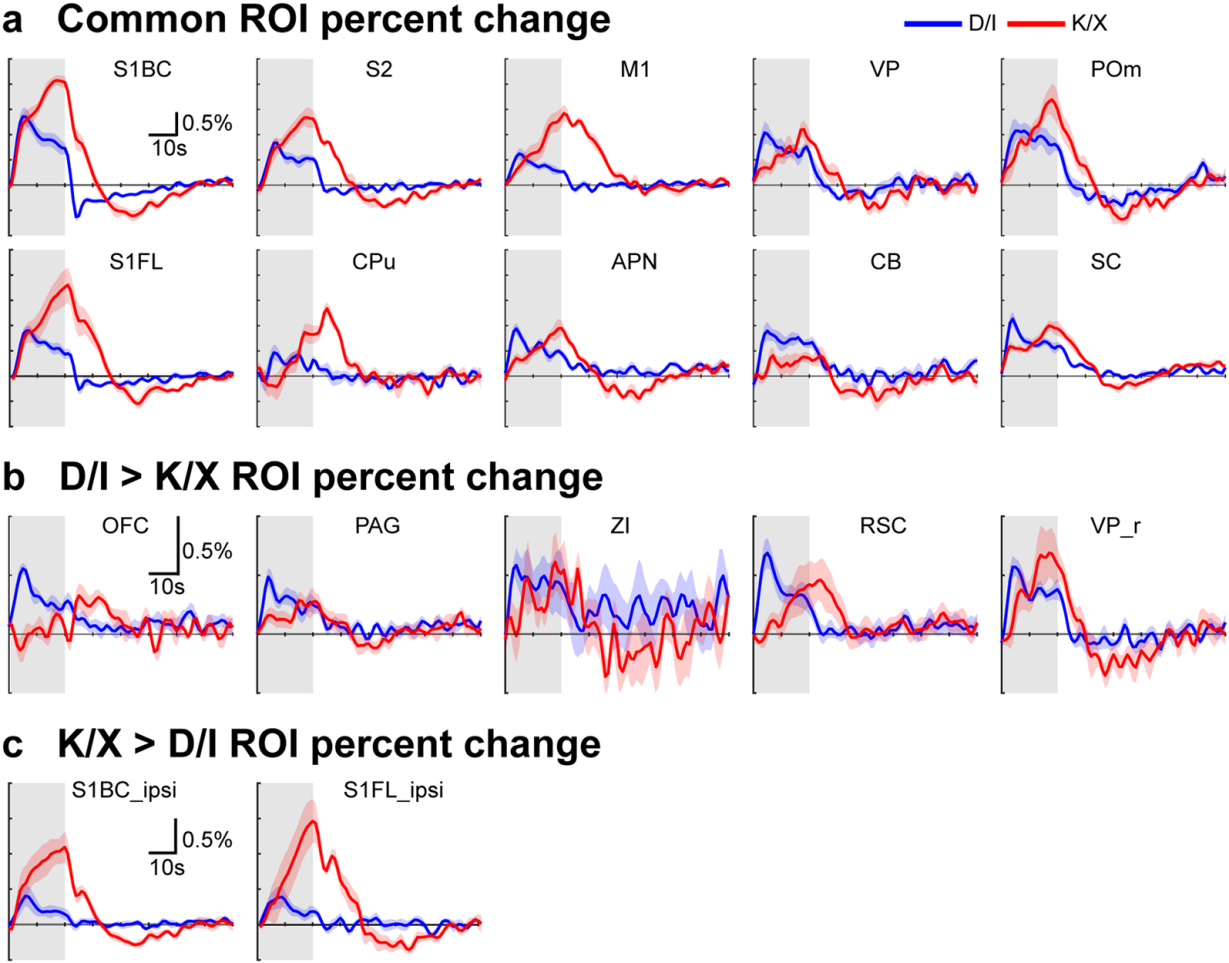
Time courses of commonly or exclusively active ROIs obtained under D/I and K/X anesthesia (**a**) ROIs common to both D/I and K/X plotted. S1BC, primary somatosensory barrel cortex; S2, secondary somatosensory cortex; M1, primary motor cortex; VP, ventral posterior thalamus; POm, posterior medial thalamus; S1FL, primary somatosensory forelimb; CPu, caudate putamen; APN, anterior pretectal nucleus; CB, cerebellum; SC, superior colliculus. Under K/X, slow BOLD increase follows the initial rapid response. (**b**) ROIs based on significant clusters found in D/I, but not in K/X plotted. OFC, orbitofrontal cortex; PAG, periaqueduct gray; ZI, zona incerta; RSC, retrosplenial cortex; VP_r, right ventral posterior thalamus. (**c**) ROIs based on significant clusters found in K/X, but not in D/I plotted. All ROIs are anatomically drawn ROIs based on the CCFv3 atlas.

To determine similarity and difference of activated functional sites by two different anesthetics, all fMRI runs in the 8-hour experiment were averaged and one-sample t-test activation maps were obtained via general linear model (GLM) (Fig. 4a). Note that two-sample t-test maps were not used due to differing HRF shapes used for GLM and large differences in sensitivities, which may underrepresent D/I maps with the current threshold used. Group activation map under D/I shows widespread thalamic clusters along with several other clusters not present in K/X: orbital frontal cortex (OFC), retrosplenial cortex (RSC), zona incerta (ZI), subiculum (S), periaqueduct gray (PAG), and cerebellum (CB) (Fig 4a). Under K/X, bilateral cortical clusters are seen. For easier comparison, group activation maps were binarized and composited to easily distinguish ROIs common and exclusive to both anesthetics (Fig. 4c). The composite maps were not cluster-corrected, so some clusters that were corrected for under D/I or K/X appear on the binarized map (e.g., CB). Common ROIs were limited to the main whisker and forepaw somatosensory axis: anterior pretectal nucleus (APN), ventral thalamus (VP), POm, S1BC, S1FL, caudate putamen (CPu), superior colliculus (SC), M1, and secondary motor cortex (M2). Anatomical ROIs were used to extract t-value from the common and exclusive ROIs and plotted to compare the sensitivities between anesthetics (Fig. 4d). We found significant difference between D/I and K/X in cortical sensory ROIs and right POm with K/X resulting in higher t-values. Amongst the listed D/I-exclusive ROIs, only OFC showed significant difference while ZI and PAG did not. In addition, percent change was plotted from each ROI in which all areas, except for OFC, showed response under K/X (Fig. 5a,b). D/I also presented with ipsilateral response (ipsilateral/contralateral) in S1BC (0.53/1.43%) and S1FL (0.39/0.90%), although not as sensitive as under K/X in S1BC (1.09/2.27%) and S1FL (1.46/1.80%) (Fig. 5c). With the inclusion of ZI, we have shown both anesthetics can map most of the main WP somatosensory axis (minus reticular nucleus) albeit with a difference in spread of activity, sensitivity, and cortical bilaterality.

## Discussion

In our mouse 15.2T fMRI study, we evaluated the sensitivity and stability of evoked somatosensory response under intravenous infusion of dexmedetomidine with inhaled isoflurane and ketamine/xylazine cocktail to further characterize their applicability in mouse fMRI. We found both anesthesia protocols produce stable BOLD response throughout eight hours in multiple cortical areas along with robust and expected brain-wide activation regardless the hypercapnic state with elevated pCO_2_ and depressed pH, which can reduce hemodynamic reactivity and functional response^52^. Although normal vascular physiological condition can be achieved with mechanical ventilation, as was done with resting-state fMRI^38^, we chose not to intubate to limit possible arousal and the technical difficulty of mouse intubation. Furthermore, evoked sensory studies using medetomidine/isoflurane or ketamine/xylazine in mice have adopted self-breathing state which is closer to our experiment design^6,9,34,35,40^.

HR and RR were stable for both anesthetics throughout the eight hours, although there appears to be an insignificant increase in HR under K/X. Although these values were stable, we noticed that two mice under D/I responded to toe pinch at post 4 hours during ABG measurements, but didn’t respond at post 6 hours which suggests fluctuating anesthetic depth regardless of a stable RR or HR. These mice were excluded from our reported ABG measurements. Similarly, we noticed mice were prone to RR fluctuations after four hours during fMRI sessions, unlike our bench measurements, which led us to increase isoflurane concentration to 0.5% until RR stabilized. Constant electrical stimulation along with loud acoustic MR noise may arouse the mice to an awake state which was also reported in a similar rat study under medetomidine^29^. Unlike D/I, all but one mouse under K/X were well sedated during fMRI duration. HR was higher under D/I which may be attributable to dexmedetomidine as isoflurane was found to have negligible effect on HR while both xylazine and ketamine were found to decrease HR in rats^38,53,54^. Our RR was similar to a medetomidine-only anesthetized mouse study suggesting our 0.3% isoflurane had negligible effect on RR^35^. Nonetheless, we are still able to report robust BOLD response with both anesthetics regardless of hypercapnia and depressed physiology.

Both dexmedetomidine and xylazine are vasoconstrictive α2 agonists that primarily act on the presynaptic terminals of arousal nuclei in the brainstem, which leads to a sleep-like state^26^. Dexmedetomidine is found to lead to greater sedation in rabbits and dogs, which suggests a primarily neural effect^46,47^. However, it can be assumed it may have greater vasoconstrictive effect as well. As such, we observed lower hypercapnic condition in D/I compared to K/X which may better preserve vasoconstriction under D/I. However, this may simply be a RR difference and comparison between intubated mice may be warranted to properly compare dexmedetomidine and xylazine. Ketamine is a noncompetitive NMDA antagonist that affect both excitatory and inhibitory populations in the cortex with minor effect on cerebral vasodilation^48,52,55^. Due to this, we hypothesize ketamine to cause pyramidal neuron disinhibition during our evoked studies^56^. Isoflurane is a GABA_A_ agonist which is known to strongly suppress neural activity and vasodilate vessels brain-wide^57^. The combination of K/X or D/I are used to counterbalance each drug’s effect on neural activity and vasculature. Dexmedetomidine alone was found to cause seizure-like activity in rats but was found to be abolished with the addition of 0.3% isoflurane, which we adopted^25^. The vasoconstrictive medetomidine is counterbalanced with the vasodilative isoflurane in small doses with the goal to achieve a more normal vascular state. This is similarly done with xylazine and ketamine, respectively.

The low sensitivity under D/I can most be attributed to the reduction of bottom-up processes by inhibiting the norepinephrine neurons in the locus coeruleus (LC). Due to nonuniform projection of LC neurons across the brain area, the effect of dexmedetomidine to fMRI responses may be different for sensory, visual, or olfactory stimuli as an aforementioned visual study reported robust response within 65 min^40,48^. The fast BOLD response under D/I is due to the vasoconstrictive nature of dexmedetomidine. D/I decreased baseline CBF and severely vasoconstricted (up to 50%) both arteries and veins in rats^25^. This condition results in higher vascular tone, which may explain the rapid HRF^58^ we observed under D/I which is also similarly observed in other fMRI studies utilizing medetomidine^29,32,40,59^. Low sensitivity may also be caused partly by isoflurane. However, the isoflurane utilized was 0.3%, with other studies showing dose-dependent increase in perfusion and reduced cerebrovascular reactivity starting from 0.7%^28,60^. Thus, our 0.3% isoflurane may have had negligible effect on vasoreactivity and neural response^25^.

Somatosensory fMRI response under K/X is slower and stronger than that under D/I. Self-breathing K/X resulted in 7–9% increase in artery and vein diameter when compared to mechanically intubated K/X mice^52^. Pre-vasodilated vessels may explain the rather slower rate of the HRF due to a lower vascular tone. The higher sensitivity under K/X may be due to neural effect than vascular as vasodilation of vessels should theoretically reduce the magnitude of relative changes in BOLD. As ketamine inhibits both excitatory and inhibitory neurons at anesthetic dose, evoked stimulation may activate excitatory neuronal populations with suppressed inhibitory neurons, enhancing local recurrent activities in the cortical areas^49^. Evidence for disinhibition comes when comparing cortical response between D/I and K/X. We report higher sensitivity in both the contralateral and ipsilateral cortical areas under K/X than D/I, while subcortical responses are similar between the two (Fig. 4d). In the awake state, we also reported weaker cortical V1 response compared to K/X anesthesia from visual stimulation, which is supported by findings that inhibition dominates the awake visual response^16,61^. When comparing signal change, under D/I, ipsilateral response was 37% of the contralateral response in S1BC and 43% of S1FL, while under K/X, ipsilateral response was 48% of contralateral S1BC and 81% of S1FL (Fig. 5 a,c). We believe the large S1FL ipsilateral response ratio for both anesthetics to be due to the ipsilateral S1FL area being adjacent to the (contralateral) primary mouth and nose somatosensory area. These regions have high activation which we believe is due to the electrical stimulation of the whisker-pad.

Both anesthesia protocols lead to stable BOLD response throughout eight hours except for a significant increase in sensitivity in S1BC at 6 h post-induction under D/I (Fig. 3b). Our results are consistent with a rat fMRI study showing stable S1FL BOLD response throughout 6 h from electrical FP stimulation under IV medetomidine^29^. The main differences between the two anesthetics are the initial sensitivity, cortical spread, and dynamics. We report greater initial sensitivity under K/X, with most functional clusters appearing within the first 2 h, while D/I requires 4 h and numerous averaging to reduce noise for better SNR.

We report the same observed whisker network from multi-whisker mechanical stimulation in awake mouse fMRI with differences in spread of the significant clusters and addition of several higher-order nuclei in our study^7^. Unlike the awake mice, we also found clusters in anterior pretectal nucleus (APN), superior colliculus (SC), and retrosplenial cortex (RSC). APN has been found to be involved in sensory processing, particularly in having antinociception function from peripheral noxious stimulation^63^. Since we found activity in APN from both anesthetics, activity may be response to noxious stimulus via electrical stimulation instead of disinhibition. Alternatively, APN activity may simply be due to strong recurrent projections from POm or SC^64,65^. SC has multisensory integration of sensorimotor and visual system with strong cortical connection from S1BC^66^. Similarly RSC is also functionally involved in multisensory integration and navigation and also have prominent sensory cortical connections^67^. Awake, restrained mice would most likely have feedforward/feedback modulation when whiskers are stimulated and would thus not “consciously” process information such as location of stimulus and are more prone to adaptation, while an anesthetized brain state would not have the modulation intact and would allow for unmodulated signal propagation if stimulated long enough^5,32,61^. We also report a larger spread in the thalamic areas under both anesthesia when compared to awake which may similarly be due to the loss of inhibitory modulation within the thalamocortical circuit. In addition, we show bilateral thalamic activity, which is expected due to our stimulation paradigm, yet the left thalamus contralateral to stimulated WP compared to the right thalamus contralateral to stimulated FP show a more sensitive response within the better developed whisker-somatosensory network (Fig. 4c).

Several limitations are present in this study. First, cluster activation in OFC and PAG under D/I suggests nociceptive response. As we reported fluctuating RR during fMRI scans, it is unlikely mice were fully sedated throughout the 8-h fMRI experiment. Second, by comparing both response map via GLM (Fig. 4) and signal change between D/I and K/X via ROI analysis (Fig. 5), we further validate the concern about needing proper HRF for GLM analysis^23,59^. D/I appears to have similar shapes throughout the brain which may explain the larger significant clusters in the subcortical areas, while K/X has widespread cortical, but limited subcortical clusters. When comparing HRF shape for K/X, certain areas have either slower dynamics or wider shape than the S1BC. This may underestimate the sensitivity and stability of thalamic areas under K/X as we utilized t-values from GLM analysis. As we designed the anesthesia-based HRF from the contralateral S1BC and S1FL (see Supplementary Fig. S1 online), the low clusters from GLM, but positive signal change via the ROI analysis shows the need to employ different K/X HRFs for subcortical regions. Third, we applied an unconventional stimulation paradigm of simultaneous FP and WP stimulation. This makes it difficult to compare to published studies. However, as our goal was to characterize the brain-wide BOLD response, we believe this paradigm allows for novel findings. Additional WP only stimulation will be necessary to map brain-wide response of widely used whisker stimulation without possible modulation influence from FP stimulation. Fourth, although we showed hemodynamic and physiological stability and characteristics of both anesthetics, we have no measurements of brain state to fully characterize these protocols. We have only inferred stable brain state by showing stable BOLD response along with physiology. Cortical EEG or LFP can verify the mouse brain state.

In conclusion, we have successfully demonstrated the feasibility of using both D/I and K/X for evoked BOLD fMRI in mice. We have mapped whisker- and forepaw somatosensory network that are also reported by various studies under both protocols and found differences in activated areas and HRF properties. Such differences allow for better understanding of the anesthetic effect on hemodynamics and merit further investigation such as the neural connection to the observed hemodynamics. We found D/I capable in mapping expected networks but suffer from low sensitivity which suggests prolonged experiment time is needed for complete functional studies with BOLD fMRI. Sensitivity issues may be a stimulation modality problem suggesting FP stimulation may not be an ideal benchmark for mice. K/X results in high sensitivity without causing unrelated activity other than cortical disinhibition. We suggest K/X to be great for investigating connectivity studies, but it may not be ideal for plasticity-related studies within the cortex as it may mask any small changes observable with BOLD. Overall, both anesthetic combinations are viable for mouse fMRI.

## Methods

### Animal care

All experiments were performed with approval by the Institutional Animal Care and Use Committee (IACUC) of Sungkyunkwan University in accord to standards for humane animal care from the Animal Welfare Act and the National Institutes of Health Guide for the Care and Use of Laboratory Animals. Experiments were designed and carried out in compliance with the ARRIVE guidelines. In total, twenty-six 11–12-week-old C57BL/6 mice (27–30g; Orient Bio, South Korea) were used for this study via the following experimental breakdown: 14 for ABG and 12 for fMRI with 6 animals per anesthetic group. Two mice were excluded under the dexmedetomidine/isoflurane group. Mice were grouped (4–6/cage) in standard caging under a 12 h day/night cycle with food and water provided *ad libitum*. Experimenters were not blind to the different stages of the experiments.

### Preparation and anesthesia

Mice were initially inducted with 4% isoflurane in a 20% O_2_/80% air mixture. For dexmedetomidine/isoflurane (D/I), isoflurane was reduced to 2% for maintenance while prepping, while for ketamine/xylazine (K/X), mice were given an intraperitoneal (IP) bolus injection with isoflurane discontinued. During preparation, the tail vein was cannulated with a 31G needle for intravenous (IV) administration of anesthesia via a syringe pump (Harvard Apparatus Standard Infuse/Withdraw PHD ULTRA). Mice were fixed onto a custom-built MRI cradle by securing their incisors over a bite bar and head via ear bars. Once secured, the cradle was transferred to the magnet and connected to a small animal ventilator (Model 1030, Small Animal Instrument Inc., Stony Brook, USA) for spontaneous respiration with a 20% O_2_/80% air mixture at a rate of 90 breaths/min through the cradle nose cone. Temperature was monitored with a rectal probe and maintained at 37.5°C via warm-water circulation pad. Respiration and heart rate were monitored with a pressure detector placed on the ventral surface and pulse oximeter (SA Instruments, Inc., Stony Brook, NY, USA) placed on the tail, respectively. Physiology was monitored using a data acquisition system (Acknowledge, Biopac Systems, Inc., Goleta, CA, USA).

For arterial blood gas (ABG) measurements, mice were anesthetized and prepped following the routine as described above with the addition of femoral artery catheterization before fixating onto the cradle. Hair was removed at the surgical site and sterilized before an incision was made to expose the femoral artery. The artery was clamped upstream from the site of catherization before a catheter made by a PE-10 tube was inserted into the artery. After insertion, the clamp was removed, and the catheter tied down with suture. All catheters were filled with saline.

Following preparation, D/I or K/X anesthesia was conducted via the following protocol which is also outlined in Fig. 1a:

*Dexmedetomidine/isoflurane*: After the cradle was transferred into the magnet, an IV bolus of 0.05 mg/kg of dexmedetomidine (Precedex, Hospira, NC, USA), diluted to 0.05 mg/mL, was given followed by the discontinuation of isoflurane. Ten minutes after IV bolus, IV infusion of 0.05 mg/kg/h was started. Isoflurane was continued 1 hour post IV bolus at 0.3%. Respiration and heart rate were monitored to be within 140–150 and 280–400 bpm respectively. Isoflurane was increased to 0.5% for any mouse that deviated from these ranges and put back on 0.3% once stable.

*Ketamine/xylazine*: A bolus of 100 mg/kg ketamine (Yuhan, Korea) and 10 mg/kg xylazine (Rompun, Bayer, Korea) was given IP after isoflurane induction. After 30 minutes post bolus, IV infusion of K/X was started at a dose of 45/2.25 mg/kg/h respectively, which was determined by preliminary dose-dependent studies. Respiration and heart rate were monitored to be within 180–230 and 200–300 bpm respectively. Infusion dose was increased to 50/2.5mg/kg/hr for any mouse that deviated from these ranges and put back to 45/2.25 mg/kg/hr once stable.

### Arterial blood gas measurements

ABG measurements were carried out in a separate group of mice on the bench using i-STAT CG8+ portable clinical analyzer (Abbot Point of Care, Princeton, NJ). Cartridges were filled with 95µL of blood at 1, 2, 4, 6, and 8 hours post anesthesia induction for blood gas analysis. Anesthesia depth was checked at every blood sampling via toe pinch. Two mice under D/I responded to toe pinch at the 4-hour sampling but didn’t respond at the 6-hour sampling. However, these mice were excluded from the results. Heart rate and respiration rate were also recorded at 30 min intervals during ABG measurements. All animals were sacrificed at the end of experimentation due to having sampled a total of 0.475 mL of blood.

### Stimulation parameter

For right whisker-pad (WP) electrical stimulation, a five-pin electrode pad originally designed for rat WP was adapted for mouse^68^. A 2x2 electrode array with 2 mm apart anodes and a cathode center was placed on the mouse’s right whisker-pad for stimulation. For left forepaw (FP) electrical stimulation, two 30G needle electrodes were inserted subcutaneously between the 1,2 and 3,4 digits of the left FP. Stimulation parameters were at a frequency of 4 Hz, pulse width of 0.5 ms, and current intensity of 0.4 mA for WP stimulation and 0.6 mA for FP stimulation. Stimulation parameters for FP were adapted from previous work that optimized forepaw response to IP K/X^43^, while WP stimulation parameters were optimized in a pilot study. The following block design was used for all experiments: 40s baseline – 20s stimulation – 60s interstimulus interval – 20s stimulation – 60s recovery. Stimuli were sent via a constant current isolator (ISO-Flex, AMPI, Jerusalem, Israel) driven by a pulse generator (Master 9; World Precision Instruments, Sarasota, FL, USA).

### fMRI experiments at 15.2 T

Data were acquired on a 15.2 T (Bruker BioSpec MRI, Billerica, MA, USA) equipped with a 11cm horizontal bore magnet and actively shielded 6-cm gradient. A 15mm ID surface coil was used for both transmission and reception. Mouse brain was placed at the isocenter of magnet and field inhomogeneity was minimized via MAPSHIM protocol in Paravision 6.0.1 software. After preparation and calibration, EPI acquisition started approximately 1-hour post anesthesia induction. Anatomical images were acquired using fast low angle shot (FLASH) sequence with the following parameters: matrix size = 256 x 128, field of view (FOV) = 15.80 x 7.65 mm^2^, repetition time/echo time (TR/TE) = 3000/45 ms, and number of averages = 4. Functional scans were acquired using gradient-echo echo planar imaging (EPI): matrix size = 120 x 58, FOV = 15.84 x 7.65 mm^2^ (0.13 x 0.13 mm^2^ in-plane resolution), 18 0.5-mm-thick slices, TR/TE = 1000/11.5 ms, 50° flip angle, and 10 dummy scans.

### Image preprocessing

Images were preprocessed and analyzed using Analysis of Functional NeuroImaging (AFNI, (https://afni.nimh.nih.gov/), FMRIB Software Library (FSL, https://fsl.fmrib.ox.ac.uk/fsl/fslwiki/), and SPM12 (https://www.fil.ion.ucl.ac.uk/spm/software/spm12/). All images were converted to NIFTI format, scaled up 10x and functional images were corrected for slice time (3dTshift, AFNI), motion (3dVolreg, AFNI), and linearly detrended (3dDetrend, AFNI). Brain masks were generated using bet (FSL) and the skull-stripped images were normalized to an in-house template based on the Allen Institute of Brain Science (AIBS) common coordinate framework (CCFv3) reference atlas using SPM12.

### Image analysis

Functional images were analyzed using a general linear model (GLM). GLM analysis was optimized by adapting Lambers *et al.*^59^. The stimulation paradigm was first convolved using SPM12’s canonical double gamma function and used as a regressor for the first-level analysis (3dDeconvolve, AFNI). Second-level analysis was conducted with 3dttest++ (AFNI) in which the images were smoothed by a Gaussian kernel full-width half maximum (FWHM) of 0.264 mm (2x voxel size). These initial group response maps were used to extract signal change in the activated somatosensory area under D/I and K/X to fit the canonical double gamma HRF. The somatosensory signal was normalized to its max value and was fit to the double gamma function by defining a sum of squared error in which 4 parameters [b, p1, p2, V] were estimated based on the equation (1). The parameters characterize the width and peaks of the gamma functions along with the ratio of the peak and undershoot. Canonical values are SPM default [1, 6, 16] with calculated values for D/I [1.5, 4.5, 8.5, 2] and K/X [0.5, 3, 16, 3].

1 $$h\left( t \right) = \left( {\frac{{t^{{p1 - 1}} b^{{p1}} e^{{ - bt}} }}{{\Gamma \left( {p1} \right)}} - \frac{{t^{{p2 - 1}} b^{{p2}} e^{{ - bt}} }}{{{\text{V}} \cdot \Gamma \left( {p2} \right)}}} \right)$$

All response t-value maps presented were created by using the anesthesia-specific HRF (see Supplementary Fig. S1 online) as regressors for 1st-level and 2nd-level analysis. Cluster correction of group response maps was performed by finding clusters that pass the threshold of *P* < 0.005 uncorrected and correcting them with a family-wise error correction via Monte Carlo simulation to *P* < 0.05 (3dClustSim, AFNI)^69^.

### Region of interest-based analysis

Regions of interest (ROI) were drawn based on the CCFv3 atlas. 2D masks were drawn in the respective anatomically defined ROIs in which voxels adjacent to different areas were excluded to limit partial voxel spread. Percent change calculations were calculated with unsmoothed functional images by normalizing to the first 30 seconds of baseline. All reported signal change and t-values were extracted using the anatomically drawn ROIs (3dmaskave, AFNI). Signal change were extracted from each individual mouse, averaged, and plotted as mean ± SEM. T-values were extracted from each individual mouse’s first-level analysis results using the anesthesia-based HRF (3dDeconvolve, AFNI). T-values were averaged and presented as mean ± SEM unless specified.

### Statistics

No exclusion criteria were conducted with fMRI results with all results analyzed. Data are represented as mean ± SEM unless otherwise specified. Statistical tests were done with MATLAB (MathWorks). Multiple comparisons for two-sample t-tests were false-discovery-rate (FDR) corrected using the Benjamini and Hochberg method with α = 0.05. One/Two-Way analysis of variance (ANOVA) were corrected using Tukey’s honest significant difference criterion with α = 0.05. All significances are based on p<0.05.

## Supplementary Information

Supplementary Information.

## Data Availability

The unprocessed functional and anatomical data are available from the corresponding author upon request. They will be provided in NIFTI format. The preprocessing scripts (Unix) can also be shared upon request.
