# Supplementary material for: Characterization of brain-wide somatosensory BOLD fMRI in mice under dexmedetomidine/isoflurane and ketamine/xylazine
Source: Sci Rep. 2021 Jun 23;11:13110. doi: 10.1038/s41598-021-92582-5 (PMC8222234; doi:10.1038/s41598-021-92582-5)
Supplement: Supplementary file 1 — Supplementary Information. [file 41598_2021_92582_MOESM1_ESM.pdf]

Supplementary Material

Characterization of brain-wide somatosensory BOLD fMRI in mice under dexmedetomidine/isoflurane and ketamine/xylazine

Taeyi You[1,2+], Geun Ho Im[1+], and Seong-Gi Kim[1,2,3*]

[1]Center for Neuroscience Imaging Research (CNIR), Institute for Basic Science (IBS), Suwon, 16419, South Korea
[2]Department of Biomedical Engineering, Sungkyunkwan University, Suwon, 16419, South Korea
[3]Department of Intelligent Precision Healthcare Convergence, Sungkyunkwan University, Suwon, 16419, South Korea
[+]these authors contributed equally to this work

***Corresponding author:**

Seong-Gi Kim, IBS Center for Neuroscience Imaging Research, N Center

Sungkyunkwan University, Suwon 16419, Republic of Korea.

Email: seonggikim@skku.edu

Phone number: +82-31-299-4350

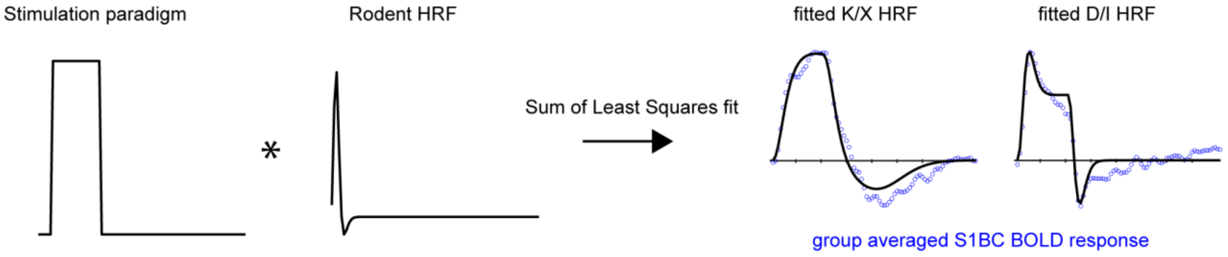

$$\text{fit(t)} = e^{-b\,t}\left(\frac{b^{\text{p1}}}{\Gamma(\text{p1})} \cdot t^{\,\text{p1}-1} - \frac{b^{\text{p2}}}{V\ \ \Gamma\ (\text{p2})} \cdot t^{\,\text{p2}-1}\right)$$

Supplementary Figure S1. Anesthesia specific HRF fitting. The canonical HRF was convoluted with the stimulation paradigm. The result was fit to the S1BC signal from either D/I or K/X. Parameters were determined via sum of least squares fit and were input into the canonical HRF to create the rodent HRF.

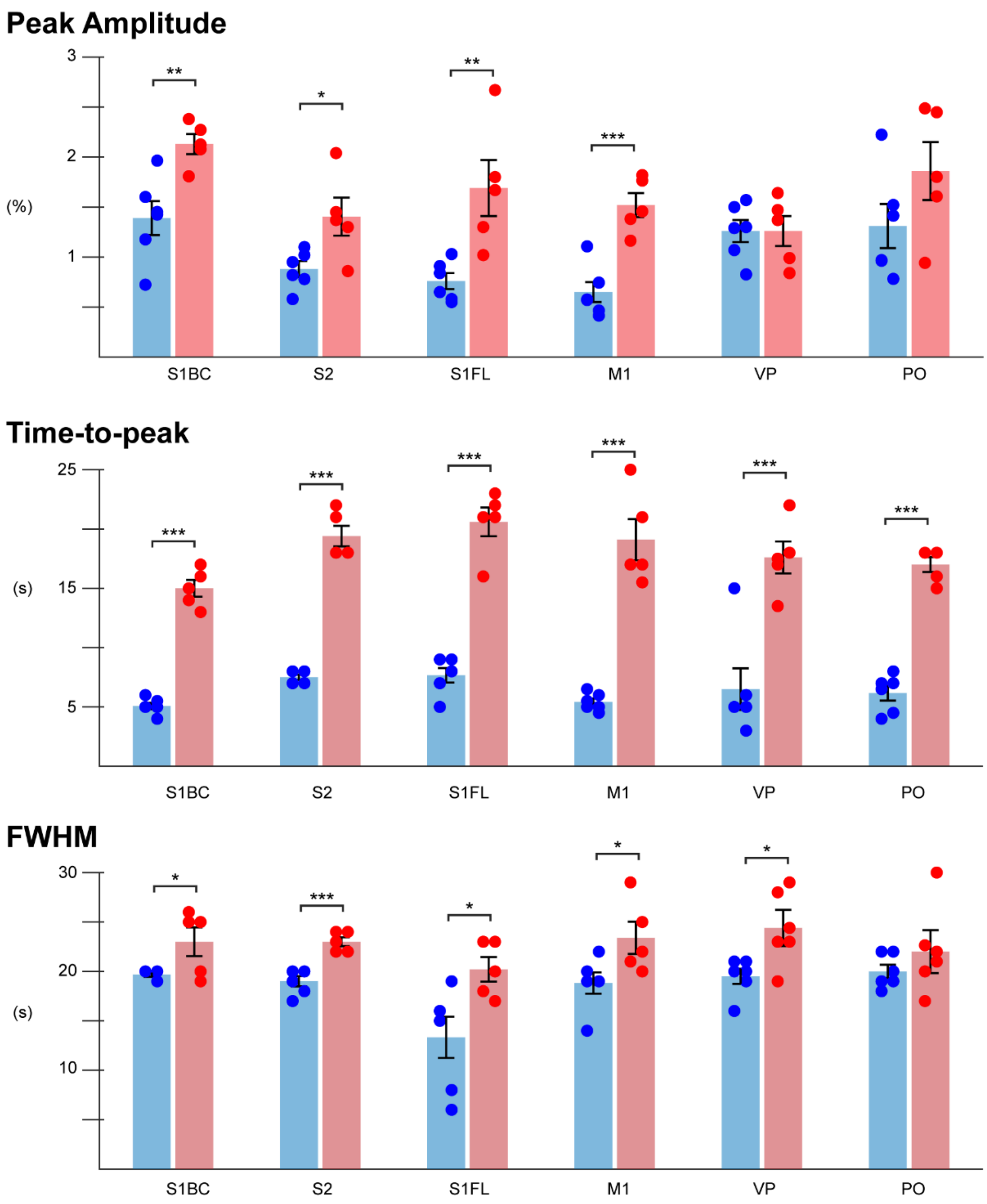

Supplementary Figure S2. HRF properties under D/I and K/X. Peak amplitude measured by finding the max value of the BOLD response during stimulation period. Time to peak calculated by finding 90% of max amplitude and estimating the time to the calculated value. Full width half maximum (FWHM) calculated between onset of curve and when curve returns to baseline. Calculations from averaged 8-hour data. Circles represent individual mouse values n=6 for both K/X (red) and D/I (blue). Corrected two-sample t-test, *P<0.05, **P<0.01, ***P<0.001
